# Supplementary material for: Sensory Health and Universal Health Coverage in Canada—An Environmental Scan
Source: Healthcare (Basel). 2024 Dec 6;12(23):2475. doi: 10.3390/healthcare12232475 (PMC11640841; doi:10.3390/healthcare12232475)
Supplement: Supplementary file 1 [file healthcare-12-02475-s001.zip › Table S1 Database searched and search terms.pdf]

Table S1: Data bases searched, and search terms.

Databases Searched	Search Terms
**PubMed (OVID)**	(((((((vision loss) OR (visual impairment)) OR (hearing loss)) OR (hearing impairment)) OR (deafblind)) OR (dual sensory loss)) OR (dual sensory impairment)) AND (((((((((universal health coverage) OR (universal health care)) OR (hearing care)) OR (hearing ADJ3 care)) OR (ear care)) OR (ear ADJ3 care)) OR (vision care)) OR (vision ADJ3 care)) OR (eye care)) OR (eye ADJ3 care))) AND ((((((((((((((((((((((((((((Canad*) OR (Ontario)) OR (Toronto)) OR (Ottawa)) OR (Quebec)) OR (Montreal)) OR (Manitoba)) OR (Winnipeg)) OR (Saskatchewan)) OR (Regina, Saskatchewan)) OR (Alberta)) OR (Calgary)) OR (British Columbia)) OR (Victoria, British Columbia)) OR (Vancouver)) OR (Newfoundland and Labrador)) OR (St John's, Newfoundland and Labrador)) OR (New Brunswick)) OR (Fredericton, New Brunswick)) OR (Prince Edward Island)) OR (Charlottetown, Prince Edward Island)) OR (Nova Scotia)) OR (Halifax, Nova Scotia)) OR (Yukon)) OR (Whitehorse, Yukon)) OR (Northwest Territories)) OR (Yellowknife, Northwest Territories)) OR (Nunavut)) OR (Iqaluit, Nunavut))
**MEDLINE (OVID)**	(((((((((((((((((((vision loss) OR (vision disorders/)) OR (blindness/)) OR (vision disorders)) OR (blindness)) OR (visual impairment)) OR (hearing loss)) OR (exp hearing loss/) OR (deafness)) OR (hearing impairment)) OR (deafblind)) OR (exp Deaf-Blind Disorders/)) OR (deaf-blind disorders)) OR (persons with hearing impairments/)) OR (persons with hearing impairments)) OR (visually impaired persons/)) OR (visually impaired persons)) OR (vision, low)) OR (vision, low/)) OR (dual sensory impairment)) AND ((((((((((((((((((((((((((((((((((((universal health coverage)) OR (universal health insurance/)) OR (universal health insurance)) OR (exp Delivery of Health Care)) OR (delivery of health care)) OR (exp Health Services Accessibility/)) OR (health services accessibility)) OR (insurance, health/ or children's health insurance program/ or for-profit insurance plans/ or health benefits plans, employee/ or insurance, vision/ or medicare/ or not-for-profit insurance plans/ or single-payer system/)) OR (insurance, health)) OR (Health Policy/)) OR (health policy)) OR (Health Care Reform/)) OR (health care reform)) OR (universal health care)) OR (Universal Health Care)/)) OR (national health programs/ or state medicine)) OR (hearing care)) OR (Hearing Aids/)) OR (hearing aids)) OR (Audiology/)) OR (audiology)) OR (Hearing/)) OR (hearing)) OR (ear care)) OR (vision care)) OR (Optometry/)) OR (optometry)) OR (eye care)) OR (Ophthalmology/)) OR (ophthalmology)) OR (Diabetic Retinopathy/)) OR (diabetic retinopathy)) OR (hearing adj3 care)) OR (ear adj3 care)) OR (vision adj3 care)) OR (eye adj3 care)) AND ((Canad*)) OR (Canada/)) OR (Ontario)) OR (Ontario/)) OR (Toronto)) OR (Ottawa)) OR (Quebec)) OR (Quebec/)) OR (Montreal)) OR (Manitoba)) OR (Manitoba/)) OR (Winnipeg)) OR (Saskatchewan)) OR (Saskatchewan/)) OR (Regina, Saskatchewan)) OR (Alberta)) OR (Alberta/) OR (Calgary)) OR (British Columbia)) OR (British Columbia/)) OR (Victoria, British Columbia)) OR (Vancouver)) OR (Newfoundland and Labrador)) OR (St John's, Newfoundland and Labrador)) OR (New Brunswick)) OR (New Brunswick/)) OR (Fredericton, New Brunswick)) OR (Prince Edward Island)) OR (Prince Edward Island/)) OR (Charlottetown, Prince Edward Island)) OR (Nova Scotia)) OR (Nova Scotia/)) OR (Halifax, Nova Scotia)) OR (Yukon)) OR (Yukon Territory/)) OR (Yukon Territory)) OR (Whitehorse, Yukon)) OR (Northwest Territories)) OR (Northwest Territories/)) OR (Yellowknife, Northwest Territories)) OR (Nunavut)) OR (Nunavut/)) OR (Iqaluit, Nunavut))
**EMBASE (OVID)**	((((((((((((((((vision loss)) OR (visual impairment/)) OR (visual impairment)) OR (hearing loss)) OR (hearing impairment/)) OR (deafblind)) OR (deafblindness/)) OR

	(deafblindness)) OR (blindness/)) OR (blindness)) OR (dual sensory loss)) OR (sensory dysfunction/)) OR (sensory dysfunction)) OR (dual sensory impairment)) OR (hearing/)) OR (hearing)) AND (((((((((((((((((((((((((((((((((((((universal health coverage)) OR (health insurance/)) OR (health insurance)) OR (universal health care)) OR (universal health care/)) OR (health care policy/)) OR (health care policy)) OR (health care delivery/)) OR (health care delivery)) OR (health care system/)) OR (health care system)) OR (health care/)) OR (health care)) OR (health care planning/)) OR (health care planning)) OR (Hearing care)) OR (hearing aid/)) OR (hearing aid)) OR (hearing disorder/)) OR (hearing disorder)) OR (hearing adj3 care)) OR (ear care)) OR (ear adj3 care)) OR (vision care)) OR (visual disorder/)) OR (visual disorder)) OR (optometry/)) OR (optometry)) OR (vision adj3 care)) OR (eye care)) OR (eye care/)) OR (ophthalmology/)) or (ophthalmology)) OR (diabetic retinopathy/)) OR (diabetic retinopathy)) OR (eye adj3 care)) AND ((Canad*)) OR (Canada/)) OR (Canada)) OR (Canadian/)) OR (Canadian)) OR (Ontario)) OR (Ontario/)) OR (Toronto)) OR (Ottawa)) OR (Quebec)) OR (Quebec/)) OR (Montreal)) OR (Manitoba)) OR (Manitoba/)) OR (Winnipeg)) OR (Saskatchewan)) OR (Saskatchewan/)) OR (Regina, Saskatchewan)) OR (Alberta)) OR (Alberta/)) OR (Calgary)) OR (British Columbia)) OR (British Columbia/)) OR (Victoria, British Columbia)) OR (Vancouver)) OR (Newfoundland and Labrador)) OR (St John's, Newfoundland and Labrador)) OR (New Brunswick)) OR (New Brunswick/)) OR (Fredericton, New Brunswick)) OR (Prince Edward Island)) OR (Prince Edward Island/)) OR (Charlottetown, Prince Edward Island)) OR (Nova Scotia)) OR (Nova Scotia/)) OR (Halifax, Nova Scotia)) OR (Yukon)) OR (Yukon/)) OR (Whitehorse, Yukon)) OR (Northwest Territories)) OR (Northwest Territories/)) OR (Yellowknife, Northwest Territories)) OR (Nunavut)) OR (Nunavut/)) OR (Iqaluit, Nunavut))
**CINAHL (Ebsco)**	((((((((((((((((((((((((((((vision loss)) OR (MH Rehabilitation OF Persons with Vision Loss)) OR (Rehabilitation OF Persons with Vision Loss)) OR ((MH Deaf-Blind Disorders)) OR (Deaf-Blind Disorders)) OR (visual impairment)) OR (MH Communication Impairment (Saba CCC))) OR (Communication Impairment (Saba CCC))) OR (hearing loss)) OR (MH Hearing Disorders)) OR (hearing disorders)) OR (MH Rehabilitation of Persons with Hearing Loss)) OR (Rehabilitation of Persons with Hearing Loss)) OR (MH Deafness)) OR (Deafness)) OR (hearing impairment)) OR (MH Hearing Disorders +)) OR (MH Hearing Screening)) OR (hearing screening)) OR (deafblind)) OR (deaf-blind)) OR (dual sensory loss)) OR (MH Hearing Loss, Functional)) OR (Hearing Loss, Functional)) OR (MH Hearing Loss, Partial)) OR (Hearing Loss, Partial)) OR (dual sensory impairment)) AND (((universal health coverage)) OR (MH Universal Health Care)) OR (Universal Health Care)) OR (MH Student Health Services)) OR (Student Health Services)) OR (MH Health Services Needs and Demand)) OR (Health Services Needs and Demand)) OR (universal health care)) OR (MH Health Care Costs)) OR (Health Care Costs)) OR (Insurance, Health)) OR (MH Tertiary Health Care)) OR (Tertiary Health Care)) OR (MH Healthcare Disparities)) OR (MH Secondary Health Care)) OR (Secondary Health Care)) OR (hearing care)) OR (MH hearing aid care (Saba CCC))) OR (Hearing Aid Care (Saba CCC))) OR (MH Hearing Aid Care)) OR (Hearing Aid Care)) OR (MH Hearing Aid Fitting)) OR (Hearing Aid Fitting)) OR (Hearing ADJ3 Care)) OR (ear care)) OR (MH Ear Care)) OR (MH Ear Care (Saba CCC))) OR (Ear Care (Saba CCC))) OR MH Ear Care (Iowa NIC))) OR (Ear Care (Iowa NIC))) OR (Ear ADJ3 Care)) OR (vision care)) OR (MH Vision Care (Saba CCC))) OR (Vision Care CCC))) OR (MH Insurance, Vision)) OR (Insurance, Vision)) OR (MH Vision Tests)) OR (Vision Tests)) OR (MH Vision Screening)) OR (Vision Screening)) OR (eye care)) OR (MH eye care)) OR (MH Eye Care (Saba CCC))) OR (Eye Care (Saba CCC))) OR (MH Eye Care (Iowa NIC))) OR (Eye Care (Iowa NIC))) AND

	(((Canad*)) OR (MH Canada)) OR (Canada)) OR ((Ontario)) OR (MH Ontario)) OR (Toronto)) OR (Ottawa)) OR (Quebec)) OR (MH Quebec)) OR (Montreal)) OR (Manitoba)) OR (MH Manitoba)) OR (Winnipeg)) OR (Saskatchewan)) OR (MH Saskatchewan)) OR (Regina, Saskatchewan)) OR (Alberta)) OR (Calgary)) OR (British Columbia)) OR (MH British Columbia)) OR (Victoria, British Columbia)) OR (Vancouver)) OR (Newfoundland and Labrador)) OR (MH Newfoundland and Labrador)) OR (Newfoundland and Labrador)) OR (MH Newfoundland)) OR (Newfoundland)) OR (St John's, Newfoundland and Labrador)) OR (New Brunswick)) OR (MH New Brunswick)) OR (Fredericton, New Brunswick)) OR (Prince Edward Island)) OR (MH Prince Edward Island)) OR (Charlottetown, Prince Edward Island)) OR (Nova Scotia)) OR (MH Nova Scotia)) OR (Halifax, Nova Scotia)) OR (Yukon)) OR (MH Yukon Territory)) OR (Whitehorse, Yukon)) OR (Northwest Territories)) OR (MH Northwest Territories)) OR (Yellowknife, Northwest Territories)) OR (Nunavut)) OR (MH Nunavut)) OR (Iqaluit, Nunavut))
**Psych Info (OVID)**	((((((((((((((((((((((((((vision loss)) OR (exp Vision Disorders/)) OR (vision disorders)) OR (exp Blind/)) OR (blind)) OR (visual impairment)) OR (hearing loss)) OR (exp Hearing Disorders/)) OR (hearing disorders)) OR (exp Deaf/)) OR (deaf)) OR (exp Hearing Aids/)) OR (hearing aids)) OR (exp Cochlear Implants/)) OR (cochlear implants)) OR (Hearing impairment)) OR (exp Audiology/)) OR (audiology)) OR (deafblind)) OR (exp Deaf Blind/)) OR (Deaf Blind)) OR (dual sensory loss)) OR (dual sensory impairment)) OR (exp Multiple Disabilities/)) OR (multiple disabilities)) AND (((((((((((((((((((((((((universal health coverage)) OR (exp Health Insurance/)) OR (health insurance)) OR (exp Health Care Services/)) OR (health care services)) OR (exp Health Care Delivery/)) OR (health care delivery)) OR (exp Health Care Policy/)) OR (health care policy)) OR (exp Health Care Costs/)) OR (health care costs)) OR (exp Health Care Reform/)) OR (health care reform)) OR (exp Health Disparities/)) OR (health disparities)) OR (universal health care)) OR (exp Health Care Utilization/)) OR (health care utilization)) OR (hearing care)) OR (hearing adj3 care)) OR (ear care)) OR (exp Primary Health Care/)) OR (primary health care)) OR (ear adj3 care)) OR (vision care)) OR (vision adj3 care)) OR (eye care)) OR (exp Ophthalmology/)) OR (ophthalmology)) OR (exp Health Screening/)) OR (health screening)) OR (exp Ophthalmologic Examination/)) OR (ophthalmologic examination)) OR (eye adj3 care)) AND (((((((((((((((((((((((((((((((Canad*)) OR (Canada)) OR (Ontario)) OR (Toronto)) OR (Ottawa)) OR (Quebec)) OR (Montreal)) OR (Manitoba)) OR (Winnipeg)) OR (Saskatchewan)) OR (Regina, Saskatchewan)) OR (Alberta)) OR (Calgary)) OR (British Columbia)) OR (Victoria, British Columbia)) OR (Vancouver)) OR (Newfoundland and Labrador)) OR (St John's, Newfoundland and Labrador)) OR (New Brunswick)) OR (Fredericton, New Brunswick)) OR (Prince Edward Island)) OR (Charlottetown, Prince Edward Island)) OR (Nova Scotia)) OR (Halifax, Nova Scotia)) OR (Yukon)) OR (Whitehorse, Yukon)) OR (Northwest Territories)) OR (Yellowknife, Northwest Territories)) OR (Nunavut)) OR (Iqaluit, Nunavut))
